# A Case Report of Atypical Migratory Shulman Syndrome

**DOI:** 10.7759/cureus.69801

**Published:** 2024-09-20

**Authors:** Carine Massih, Lea Karam, Jad Okais, Tonine Younan, Carole Kesrouani

**Affiliations:** 1 Department of Rheumatology, Université Saint-Joseph and Hôtel-Dieu de France University Hospital, Beirut, LBN; 2 Department of Radiology, Université Saint-Joseph and Hôtel-Dieu de France University Hospital, Beirut, LBN; 3 Department of Pathology, Université Saint-Joseph and Hôtel-Dieu de France University Hospital, Beirut, LBN

**Keywords:** autoimmunity, connective tissue disease, eosinophilic fasciitis, musculoskeletal, skin

## Abstract

Eosinophilic fasciitis (EF), or Shulman syndrome, is a rare connective tissue disorder characterized by symmetrical and painful swelling and with progressive thickening of the skin and soft tissues with the potential involvement of internal organs such as the pleura, pericardium, and kidneys. Patients may also present with fever, myositis, arthritis, neuropathies, and other systemic symptoms. This case report describes a unique multifocal asynchronous soft tissue involvement in Shulman syndrome in a 39-year-old patient, highlighting clinical presentation, histopathological findings, differential diagnoses, treatment modalities, and patient outcomes. Atypical migratory skin lesions must be considered in the diagnosis of EF. Timely recognition of the disease is crucial for optimal treatment and better patient outcomes.

## Introduction

In 1974, Shulman et al. reported two patients with scleroderma-like skin changes, distinguishing this condition from progressive systemic sclerosis due to the absence of visceral involvement, Raynaud's phenomenon, and a favorable response to corticosteroid therapy [1]. In this paper, we report a case of unusual migratory, asynchronous, and asymmetric skin and soft tissue lesions in eosinophilic fasciitis (EF), a disease that is generally characterized by symmetric and fixed skin induration, and swelling of the extremities.

## Case presentation

**Figure 1:**
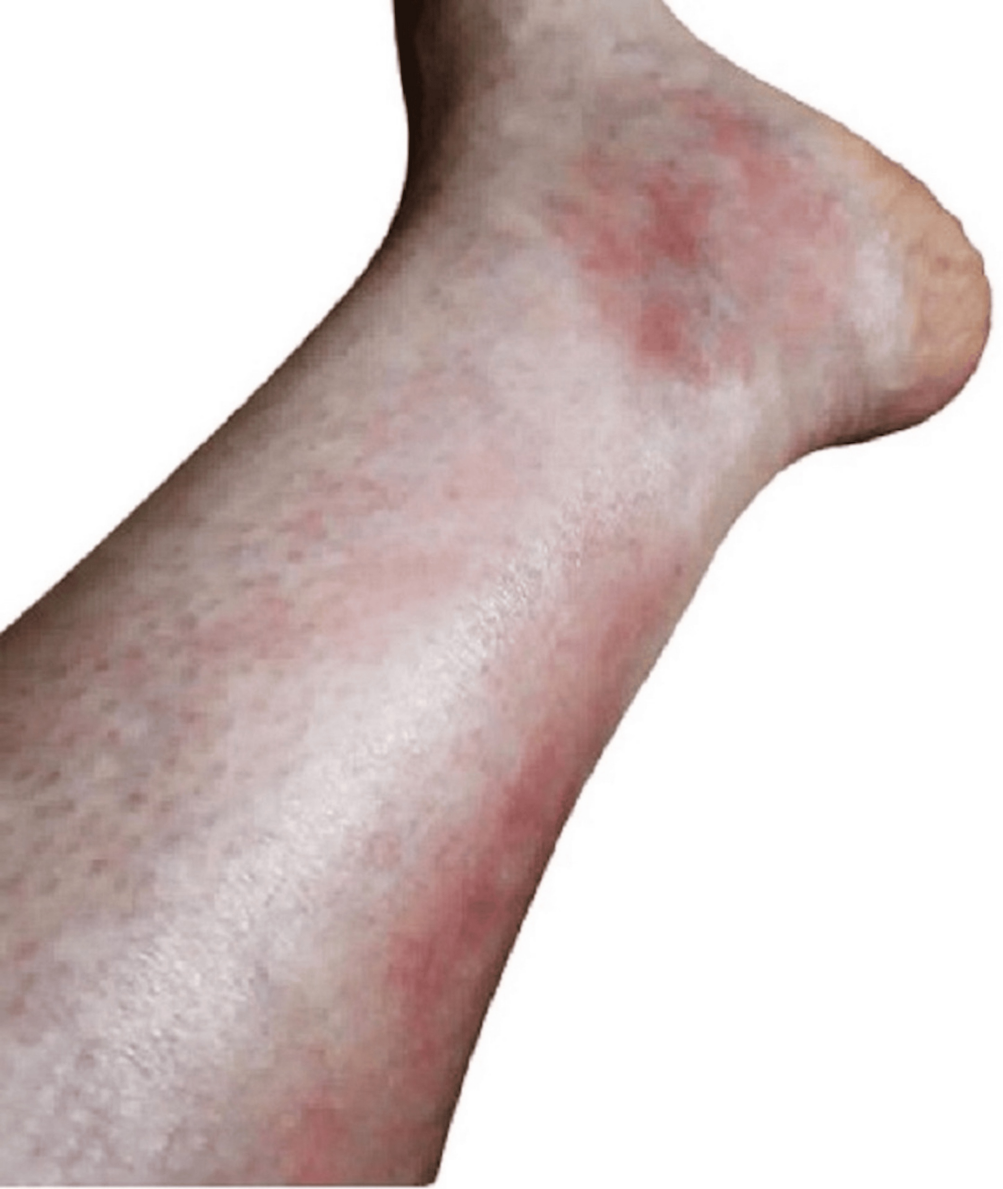
Scattered erythematous skin lesions across the lower limb

This is a case of a 39-year-old female who presented with a short history of fever and swelling, along with red, indurated, and painful skin lesions initially localized in the right lower limb (Figure 1). These lesions spontaneously resolved after 15 days. Subsequently, other skin lesions appeared in an asymmetric and asynchronous pattern, spreading to involve the left lower limb and later the left forearm. Over time, the lesions evolved into desquamating plaques. Upon clinical examination, irregular, erythematous, and indurated skin lesions were observed. Nevertheless, a highly specific clinical finding known as the 'groove sign' was notably absent.

**Figure 2:**
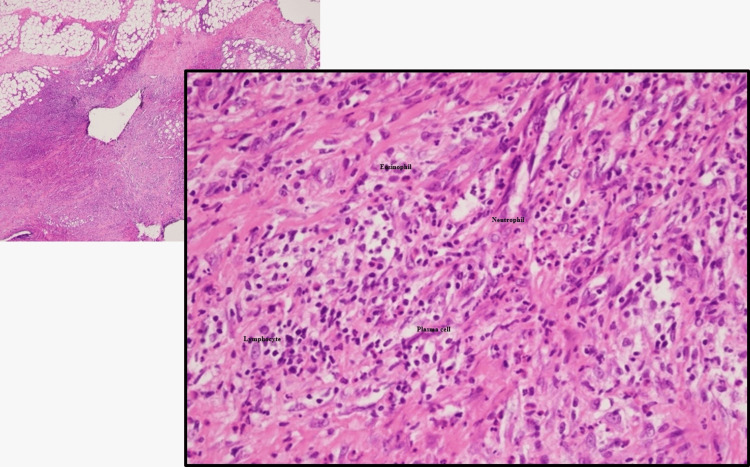
Fibrosis and numerous eosinophils among the inflammatory infiltrate of the fascia and hypodermis

A deep skin biopsy revealed fibrosis of the hypodermis and fascia, with inflammatory infiltrates including lymphocytes, plasma cells, neutrophils, and numerous eosinophils, consistent with the diagnosis of EF (Figure 2).

**Figure 3:**
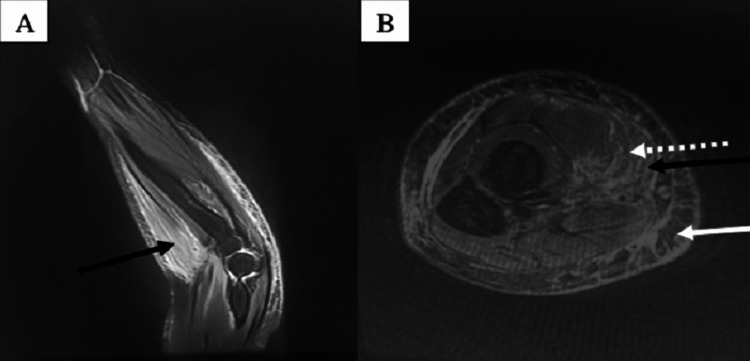
The MRI of the left forearm A: Proton density (PD) fat-saturated sequence, sagittal view; B: T1-weighted fat-saturated post-gadolinium injection sequence, axial view, showing myositis (black solid arrow) and fasciitis (white dotted arrow) affecting the anterior compartment of the left forearm and edema of the subcutaneous tissues (white solid arrow)

Extensive laboratory tests were conducted to exclude infectious, autoimmune, and paraneoplastic etiologies. The Wright and Widal tests, serology for rickettsiae and borrelia, antinuclear antibody (ANA), and extractable nuclear antigen (ENA) tests were negative, but erythrocyte sedimentation rate (ESR) and C-reactive protein (CRP) were elevated. The eosinophil levels were within the normal range. Polyclonal paraproteinemia was detected through serum protein electrophoresis. The chest, abdominal, and pelvic CT scans showed no abnormalities. An MRI of the left forearm showed myositis affecting the long supinator muscle (Figure 3 A), as well as fasciitis of the left forearm (Figure 3 B).

**Figure 4:**
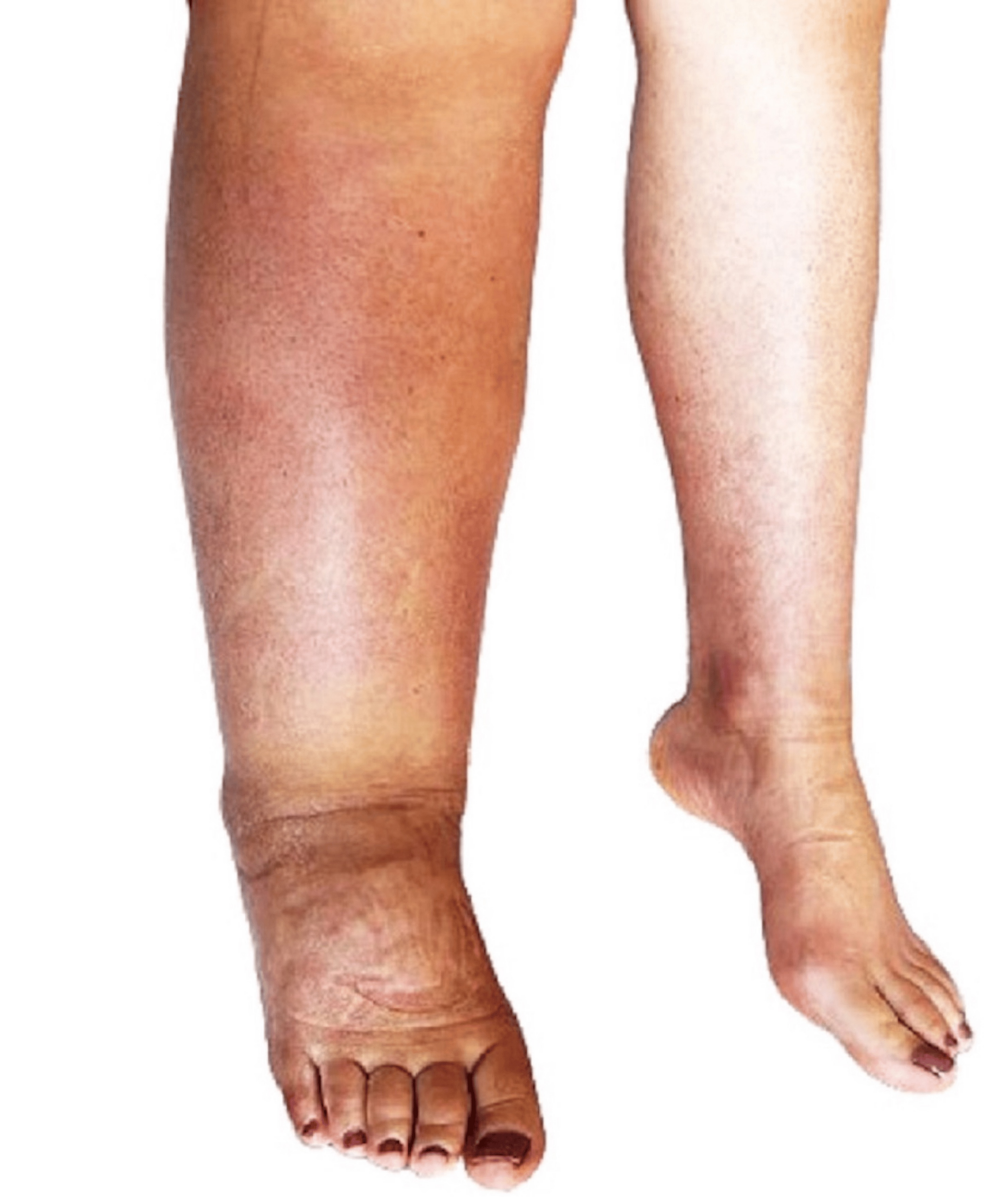
Secondary elephantiasis and relapse of EF identified by an erythematous indurated lesion over the right lower limb EF: Eosinophilic fasciitis

**Figure 5:**
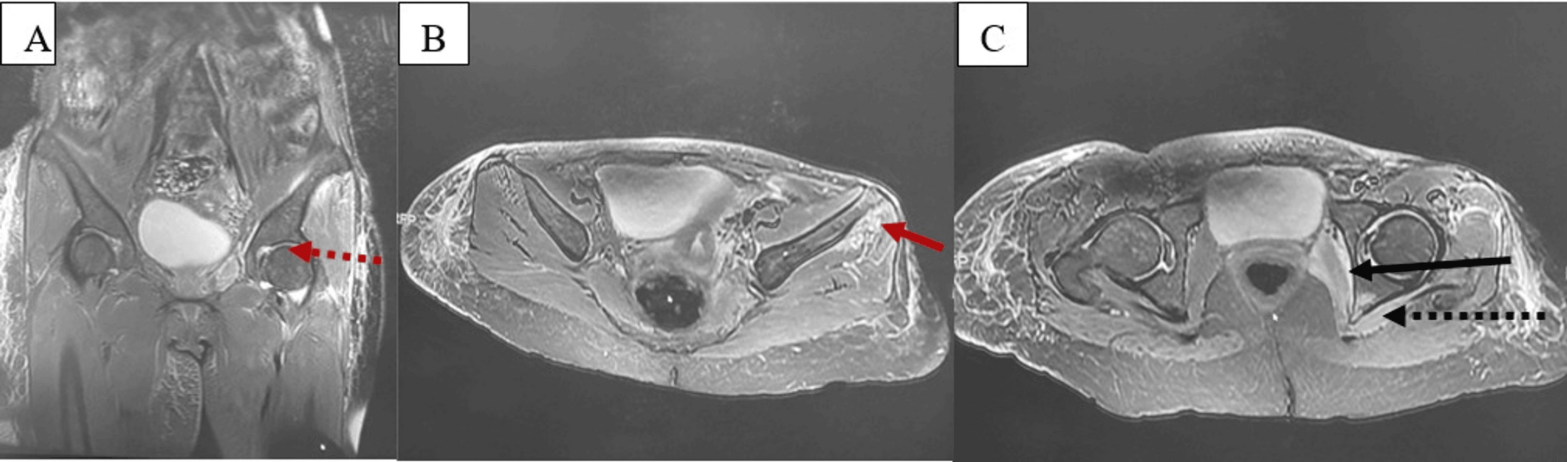
Pelvic MRI, T2-weighted sequences A: Left coxofemoral synovitis (red dotted arrow); B: Myositis of the left gluteus minimus (red solid arrow); C: Myositis of the left obturators (black solid arrow) and pelvitrochanteric muscles (black dotted arrow)

The patient was initially started on oral corticosteroids at a dose of 1 mg/kg per day, which was tapered over seven months as needed. Methotrexate at a dose of 15 mg/week was added after one month due to the occurrence of new skin lesions, despite an increase in corticosteroid therapy to 1.5 mg/kg per day. Gradual improvement in skin lesions and pain relief were noted after five months. A residual right lower limb elephantiasis persisted. A relapse occurred one year later, characterized again by multifocal migrating dermohypodermal lesions (Figure 4) and left coxofemoral synovitis (Figure 5 A), along with contiguous myositis (Figure 5 B and C), requiring treatment with 750 mg of intravenous methylprednisolone therapy for three days and a dose increase in methotrexate to 20 mg/week.

## Discussion

In 1974, Shulman described a rare scleroderma-like syndrome designated as EF. Eosinophilic fasciitis is more prevalent in Caucasian populations, affecting both sexes equally between 40 and 50 years of age [2]. Several factors that can trigger EF include autoimmune diseases such as Sjögren's syndrome, systemic lupus erythematosus, primary biliary cirrhosis, and Hashimoto's or Graves' disease [1,2]. Other reported causes of EF are the initiation of hemodialysis, physical factors such as radiotherapy and burns, severe trauma or exercise, and the use of some drugs such as statins, phenytoin, and subcutaneous heparin [1-4]. Eosinophilic fasciitis has also been associated with infectious agents such as intestinal parasitosis [5], bacterial infections with *Mycoplasma arginini* and *Borrelia burgdorferi* [6,7], solid neoplasms, as well as hematological disorders, which occur in up to 10% of patients, such as lymphomas, leukemias, allogeneic stem cell transplantation, and graft-versus-host disease [8,9].

Patients present with myalgia (67%), asthenia (38%), weight loss (26%), and, less frequently, fever [10]. Skin involvement accounts for 90% of cases and typically begins with symmetrical erythema, edema, and thickening of the skin, most frequently involving the upper limbs, followed by the lower limbs and, less commonly, the trunk and neck. However, unilateral skin lesions have been reported [11]. The 'orange peel' skin aspect and induration appear subsequently. A pathognomonic clinical sign of EF, the 'groove sign,' is characterized by a linear depression along the superficial veins of the distal limbs and can be best visualized upon limb elevation [12]. 

Other extracutaneous manifestations of the disease include arthralgia, arthritis, joint contractures, and carpal tunnel syndrome [2,13], with myositis occurring infrequently [14]. Rare presentations include involvement of the renal, pleural, and pulmonary systems, along with pericardial effusion [4,15]. Some patients may develop vascular complications such as digital gangrene [16].

This case report describes an atypical presentation of Shulman syndrome, where the patient exhibited a migrating pattern of skin and soft tissue lesions. In previously published cases, only gradual and persistent skin involvement was described [17]. Thus, a comprehensive analysis of the patient's clinical presentation, laboratory, imaging, and pathology findings is recommended for diagnosing this disease. Laboratory testing often reveals peripheral eosinophilia, although it is not a definitive factor for diagnosis or prognosis. Elevated acute-phase reactants and hypergammaglobulinemia may also be present. Antinuclear antibodies may be positive, usually with a negative anti-double-stranded DNA test and extractable nuclear antigen panel [2,4].

The MRI is an important diagnostic and prognostic tool for EF, showing increased signal intensity within the fascia [18]. The definitive, though not essential, tool for diagnosing EF is skin and muscle fascia biopsies, as fibrosis spreads from the subcutaneous tissue and fascia to the deep layers of the dermis. In the initial stage of the disease, characteristic pathohistological findings are edema in the soft tissue and fascia and inflammatory infiltrate consisting of lymphocytes, plasma cells, histiocytes, and eosinophils. In some cases, the infiltration can only be localized and transient. As the disease progresses, atrophy of the epidermis and thickening of the fascia are reported on pathologic examination [19]. 

Due to the rarity of the disease and the absence of standardized treatment criteria or uniform clinical responses, there is no consensual therapeutic strategy. In rare cases, some patients may experience spontaneous improvement without treatment [4]. Early initiation of treatment is crucial to preserve joint mobility, with systemic steroids being the first-line therapy, typically starting at 1 mg/kg per day followed by gradual tapering [1,4]. Between 70% and 90% of patients show a partial to complete response [4], although softening of the affected skin can take weeks to months [1]. Methylprednisolone pulses have shown effectiveness, particularly when used early in treatment [20]. Poor prognostic factors include lack of methylprednisolone pulses at treatment initiation, a diagnostic delay of over six months, trunk involvement, age under 12 years, and the presence of morphea-like skin lesions or dermal fibrosclerosis on histopathology [4].

Second-line treatments warranted by either failure or dependency on high-dose corticosteroids primarily include methotrexate. Other options based on case reports or case series include D-penicillamine, cyclosporine, azathioprine, mycophenolate mofetil, cyclophosphamide, hydroxychloroquine, immunoglobulin, tocilizumab, rituximab, dapsone, inhibitors of tumor necrosis factor such as infliximab, psoralen-ultraviolet A bath photochemotherapy, and extracorporeal photochemotherapy [1,4]. 

## Conclusions

This case underscores the importance of considering Shulman syndrome in patients presenting with multifocal, migrating, erythematous, and indurated dermohypodermal lesions. This pattern of skin lesions in EF has never been reported in the literature; only gradual and persistent skin involvement has been described. Although EF is a rare condition, it presents significant challenges in clinical practice due to the variability in patient presentation, underlying causes, and responses to treatment. Timely recognition and appropriate management are essential for achieving favorable outcomes in these patients.
